# Research on the evolution and improvement of China’s shadow education governance policy from the perspective of multiple streams theory

**DOI:** 10.3389/fpsyg.2023.1013243

**Published:** 2023-03-13

**Authors:** Kemi Xiang, Jinsi Liu, Xinzhong Chen, Anlu Zhang

**Affiliations:** ^1^College of Public Administration, Huazhong Agricultural University, Wuhan, China; ^2^School of Political Science and Public Administration, Wuhan University, Wuhan, China; ^3^Institute of Education Sciences, Wuhan University, Wuhan, China

**Keywords:** double reduction, shadow education, multi-stream theory, educational governance policy, off-campus training

## Abstract

From a global perspective, after-school tutoring education, also known as shadow education, has developed rapidly since the beginning of this century. However, shadow education has also brought many practical problems, such as the increased burden on parents and children, and the unfairness in education. At present, the Chinese government is vigorously implementing the double reduction policy and has achieved remarkable practical results. This study focuses on the evolution of the government policy of shadow education in China. First, it analyzed the four stages of shadow education governance policy experience: the acquiescent survival stage, the encouraging development stage, the preliminary regulation stage, and the comprehensive rectification stage. Python was used for text mining the policies from different periods and analyzing the focus of the policies in different stages by obtaining high-frequency vocabulary. Then, the multiple streams theory was used to explore the policy evolution process and change mechanism. Finally, relevant recommendations have been discussed to address the gaps in the current shadow education governance policies. The study found that the objectives, scope of adjustment, and protection of rights and interests of China’s shadow education governance policies have undergone significant changes over time. Through the constant interaction and interweaving of the stream of problems, the stream of politics, and the stream of policy, the window of opportunity for policy change was jointly promoted. The innovations of this article mainly include the following: First, the evolution of China’s shadow education governance policies was systematically reviewed using text mining methods to compare the differences of governance policies at different stages and second, multiple streams theory was used as the theoretical framework to analyze the reasons for the focus of shadow education governance policy changes.

## 1. Introduction

Shadow education, also known as off-campus training or off-campus tutoring, takes place outside of mainstream school education focused on the school subjects and charges a fee (Stevenson and Baker, 1992). Shadow education exists globally, especially in many East Asian countries (Manzon and Areepattamannil, 2014). The development of shadow education has a wide range of historical origins. For instance, in the middle of the 19th century, Russian students had a private tutor and by the end of the 19th century, tutoring institutions appeared. Around the 1980s, the topic of shadow education was published in the China Youth Daily (Zhang and Bray, 2020). The focus of shadow education policies in China is different in different periods. At present, it is guided by the “dual reduction” policy and adopts strict control of shadow education. Japan stipulates that the tuition institution is a service-oriented industry and must not be subject to the constraints of economic laws and regulations and must not endanger the rights of consumers. South Korea has included tuition education within the public education system. Different countries have adopted specific policies and regulations on shadow education. As a kind of extracurricular tutoring, shadow education has three characteristics: First, it is a supplementary tutoring education, tutoring for subjects that are taught in school. Second, it is private and is provided by enterprises or individuals for profit rather than by public education funds or voluntary tutoring. Third, it focuses on academic subjects and tuition for cultural courses such as Chinese, Mathematics, and English, and not art subjects (Postlethwaite, 2000). However, with the development of shadow education, this definition cannot completely summarize the changes and differences in shadow education. However, the mainstream education system is still accompanied by the important role played by shadow education (Ghosh and Bray, 2020). Therefore, this study mainly discusses the extracurricular tutoring and extramural training that exist outside mainstream education in schools. With the development of China’s economy and the improvement of society and culture, China’s education continues to grow and develop, and so does shadow education alongside it (Zhang and Liu, 2016). With the expansion of off-campus training institutions, it is of great importance to systematically examine and carry out an in-depth analysis of the evolution of China’s shadow education governance policies, clarify what shadow education governance policies are and why they have changed over time, and discuss how to further improve them based on the practice of educational development in the new era. This will help to better solve the problems and challenges in China’s shadow education governance, promote equity in education, and provide a reference for other countries to formulate shadow education policies.

To strengthen the main position of school education and promote the healthy growth and comprehensive development of primary and secondary school students, China has successively introduced several “double reduction” policies for the governance of off-campus training institutions since 2018 (Xue and Li, 2022). At the end of the same year, the Ministry of Education announced the progress of the special governance of off-campus training institutions across the country. In 2019, the number of off-campus training institutions continued to grow on a large scale. To further standardize the off-campus training market, the Ministry of Education issued the “double reduction” policy again in July 2021, and a new round of special rectification work for off-campus training institutions was introduced (Eryong and Li, 2022). With the closure of many off-campus training institutions as a result, the special governance of off-campus tutoring agencies achieved preliminary results. To some extent, it reduced the recourses in the market and has been supported by the public.

China’s double-reduction policy stems from the specific content and expression of the current shadow education governance policy. However, the focus of the governance policies on shadow education is different in different periods. The objective of this study is to investigate the evolution and change in China’s shadow education governance policies. It analyzes the reasons for the implementation of the current double-reduction policy, which has tightened the governance of shadow education, effectively curbed the development of extracurricular counseling in education, and reduced the burden of off-campus training to a certain extent. As long as the current evaluation system remains unchanged and entrance examinations still exist, the demand for shadow education will not disappear, and parents’ anxiety about their children’s education will persist. As the supply of off-campus training in the market dwindles, the export of tutoring demand will turn to “one-to-one” or other forms of tutoring. At present, there are many studies on shadow education, and scholars have deduced the achievements and limitations of shadow education research using bibliometric methods (Hajar and Karakus, 2022). However, the field of shadow education research is still nascent, and the terms and parameters of its research are unclear (Bray, 2010). Therefore, it is necessary to determine the reasons for the emergence of shadow education, the factors that affect the development of shadow education, as well as the consequences of shadow education, and the methods that should be adopted to deal with the problems caused by shadow education.

## 2. Literature review

With the development of society, shadow education has grown continuously, and after-school tutoring institutions have become more established and have been accepted by more people, especially by parents who are willing to invest in their children’s education. At the same time, shadow education has also impacted society in many ways, to which more and more attention is being paid in recent times. Equally, many scholars have begun to study shadow education. From a global perspective, scholars have found that there are differences in access to shadow education in different countries, and the main reason is the large socioeconomic gap (Entrich, 2020). Shadow education is parents’ investment in children’s education, but counseling does not necessarily work. On the contrary, it wastes social resources to a certain extent (Bray, 2014). In addition to domestic shadow education, there are also transnational shadow education models that families from higher social status or developed areas are more likely to use (Byun et al., 2018). The reason behind this is the differences in parents’ education and income, which directly affect their investment in shadow education (Jansen et al., 2021). Compared with China’s emphasis on competition and achievement models which sustains the prevalence of shadow education, Danish families are unwilling to use shadow education to gain advantages in an equal society (Zhang, 2021).

In the research on the factors influencing the development of shadow education, a study found that the peer effect was conducive to the popularization of shadow education (Pan et al., 2022). Shadow education has resulted in converting unpaid education into a private education charging model with some formal teachers also joining shadow education and training. In particular, studying the development of Japan’s shadow education industry, it can be concluded that shadow education will gradually be standardized and institutionalized (Mori and Baker, 2010). The socioeconomic composition of schools affects participation in shadow education. For instance, students in schools with high socioeconomic status are more likely to seek shadow education courses than those in low-status schools (Matsuoka, 2015). At the same time, in the research on the impact of shadow education, shadow education, as a unique form of privatization, directly affects the fairness and justice of society (Bray and Kwo, 2013). The reason is that students from advantaged backgrounds are more involved in shadow education than the disadvantaged (Zwier et al., 2020). However, research also reveals that children from unfavorable social backgrounds will not choose extracurricular tutoring education, which also shows that the existence of shadow education will lead to unfairness (Choi and Park, 2016). In addition, scholars have found that shadow education has a positive impact only on the performance of high-achieving (rather than low-achieving) students (Loyalka and Zakharov, 2016). Judging from the example of shadow education development in Myanmar, its development made teachers in public schools spend more time on extracurricular tutoring, thereby reducing their investment in public school education and bringing about a greater negative impact (Liu and Bray, 2020). From a wider perspective, shadow education has an impact on the larger ecosystem of education such as family and society (Luo and Chan, 2022).

This article, therefore, mainly studies the evolution of China’s shadow education governance policy and uses the multiple streams theory to discuss the changes in China’s shadow education governance policy. The multiple streams theory was proposed by American political scientist John W. Kingdon in his representative work “Agenda, Filing Program and Public Policy.” Through extensive and in-depth empirical investigations and case studies, the core links of public policy, namely the agenda establishment, and the formation of public policy have been scientifically discussed (Kingdon and Stano, 1984). Kingdon believes that the establishment of the agenda, the generation of alternatives, and the establishment of public policies are driven by the convergence of three streams: the stream of problem, the stream of policy, and the stream of politics. Generally, these three streams operate relatively independently. However, at a critical moment, policy entrepreneurs push the three streams together to open a specific “policy window,” and the three work together to achieve policy changes.

The use of the multiple streams theory is to pay attention to the changes in a certain field of policies and to identify which institutions attention should be paid to when the agenda is being finalized (Bolukbasi and Yildirim, 2022). Taking European natural gas supervision as an example, scholars used the multiple streams method to explain the setting of the European agenda to study the conditions of opening the policy window (Herweg, 2016). Although this method is questioned for rigor and experience, the key variables of the multiple streams theory interact with the characteristics of the collaborative mechanism environment of the process so that the specific case-driving factors of policy changes can be determined (Koebele, 2021). In recent years, scholars have used the theory of multiple streams theory in large-scale policy environments, mainly because they have two contributions. One is that it has promoted the development of evolutionary policies such as interruption and balance, and the other is that it has spawned large and focused scientific research (Cairney and Jones, 2016). This theory has certain relevance for the research on the transition of Chinese public policy (Howlett, 2019). It has also been widely used in the study of educational policy, but few studies have been conducted to analyze China’s current shadow education governance. Using the multiple streams theory, this study explored the mechanism and optimization direction of its evolutionary process from the perspective of the policy process of shadow education governance by focusing on the problems, policies, and political streams with the belief that this research may provide new ideas for shadow education system governance and education reform.

## 3. Historical development of the double reduction policy in China

For the research on China’s shadow education governance policy, the policy data was obtained from the website of “China Bailu Think Tank^1^”. A total of 44 policy texts were selected (Table 1). The data covered all policies on shadow education governance since 1978 that could represent the changes in China’s shadow education governance policy; in general, 10 years is regarded as a relatively long period to study the regularity of policies. During the period from 1978 to 2010, the number of policies relating to shadow education governance gradually decreased, and since 2011, their numbers have risen sharply. A total of nine policies were released in only 2 years from 2021 to 2022. This is in line with the fact that the Chinese government has intensified its governance of shadow education in recent years. Over time, it also gradually formed a more systematic way for the promulgation of policies and regulations. In addition, from the point of view of the decision-making departments that issued policies, the Ministry of Education is the main department. This indicates that the Ministry of Education is the direct leading department concerned with shadow education and has promulgated more comprehensive and detailed policies.

**Table 1:** Time and department statistics of policy release.

Release time	Number of policies	Decision-making department	Number of policies
1978–1990	8	Ministry of Education	30
		Council of State Governments	6
1991–2000	4	National People’s Congress	3
2001–2010	2	National Educational Commission	3
2011–2020	21	National Education Reform Office	3
2021–2022	9	Chinese Society of Education	1
		Ministry of Justice	1

**Figure 1:**
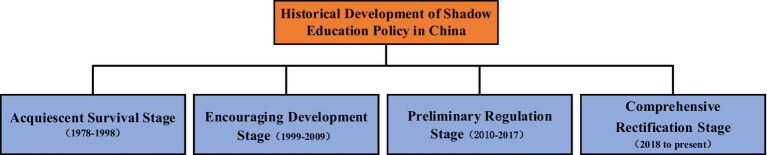
Historical changes in shadow education in China.

This study analyzes the Chinese shadow education governance policy in stages and mainly interprets the content of the policy, especially the new policies introduced in special periods to distinguish the policies in other periods. To understand the focus of policies in different periods, the best way is to find high-frequency vocabulary appearing in the policy text. Therefore, the policies introduced in different periods were analyzed using text mining. Python software was used to identify high-frequency vocabulary appearing in the texts. The relevant full-text policies from different periods were compared with each other to highlight the changes in the focus of the policies in different periods. After reading the 44 policy texts and cross-referencing them to the economic and social background of the country during different periods, China’s shadow education governance policy was divided into four stages. The first stage: at the starting stage of China’s reform and opening up the urgent task in the field of education was to restore and rebuild the system, develop education, and cultivate urgently needed specialized talents for national construction. The second stage: during this period, the country’s educational resources were insufficient, and shadow education existed in the form of tutoring, which was in its infancy. With the rapid development of China’s economy, the decision to comprehensively promote quality education was taken in 1999. It encouraged schools to be run in various forms, and shadow education was encouraged and developed rapidly during this period. The third stage: the Outline of the National Medium and Long Term Education Reform and Development Plan (2010–2020) was proposed to “regulate various social tutoring institutions and the teaching aid market.” The management of education and training institutions was preliminarily standardized. The fourth stage: at the beginning of 2018, with the introduction of a series of governance policies for off-campus training institutions, China’s shadow education governance entered a stage of comprehensive rectification. Figure 1 illustrates the four stages of the process of shadow education governance.

### 3.1. Acquiescent survival stage (1978–1998)

With the end of the Cultural Revolution, the order in the field of education was gradually restored, and “respect for knowledge and talents” was advocated. A competitive environment was re-established with examinations as an important selection mechanism and learning ability as the main criterion. The national unified examination system for enrollment in ordinary colleges and universities was restored and the system of key universities and primary and secondary schools was recovered. Families with financial ability but not enough energy after the reform and opening up had a strong demand for tutoring. Under such circumstances, the tutoring market emerged at the right moment. The government and society were acquiescent to its existence. To enter key schools to obtain a high-quality education, the competition for further education and school selection was fierce. During this period, shadow education sprouted in the form of in-school tutoring and private tutors. The policy texts of this period were entered into the Python software to obtain the high-frequency words that appeared in this period (Table 2). Judging from the policy texts, there were “Regulations,” “Teachers,” and “Teaching” that appeared more frequently. Focusing on the teaching of school teachers, the words were “Overweight,” “Relieving,” and “Schoolwork Burden,” which indicated that the main goal was to reduce students’ burden, whereby intensive supplementary lessons were not allowed and extracurricular homework was to be assigned reasonably.

**Table 2:** Statistics of the high-frequency vocabulary of policies in the acquiescent survival stage.

High-frequency words	Numbers	High-frequency words	Numbers	High-frequency words	Numbers
Regulations	36	Examination	30	Relieving	24
Teachers	35	Thought	29	Schoolwork burden	24
Overweight	33	Labor	26	Teaching	23

In the early days of the reform and opening up, there was a serious shortage of educational resources and government finances were insufficient. The key school system was a special policy adopted to improve the quality of education in line with the development of the social and economic conditions at that time (Zhang and Bray, 2017). Problems such as “entering a higher school fever” “school choice fever,” and “high fees” became more and more obvious. The government issued several policies to reduce the burden of schoolwork in primary and secondary schools. The State Education Commission issued the “Regulations on Alleviating the Excessive Academic Burden of Primary and Secondary School Students” in 1988, reiterated it in 1990, and then issued the “Opinions on Comprehensively Implementing the Education Policy and Alleviating the Excessive Academic Burden of Primary and Secondary School Students” in 1993, which required the implementation of the curriculum teaching plans for primary and secondary schools as per the new regulations. It did not allow occupying spare time or holidays to give students collective time for make-up classes. During this period, the government policy focused on regulating the educational order of primary and secondary schools and reducing the academic burden in schools. The government made clear regulations on study time and the number of homework assignments for primary and secondary school students.

### 3.2. Encouraging development stage (1999–2009)

**Figure 2:**
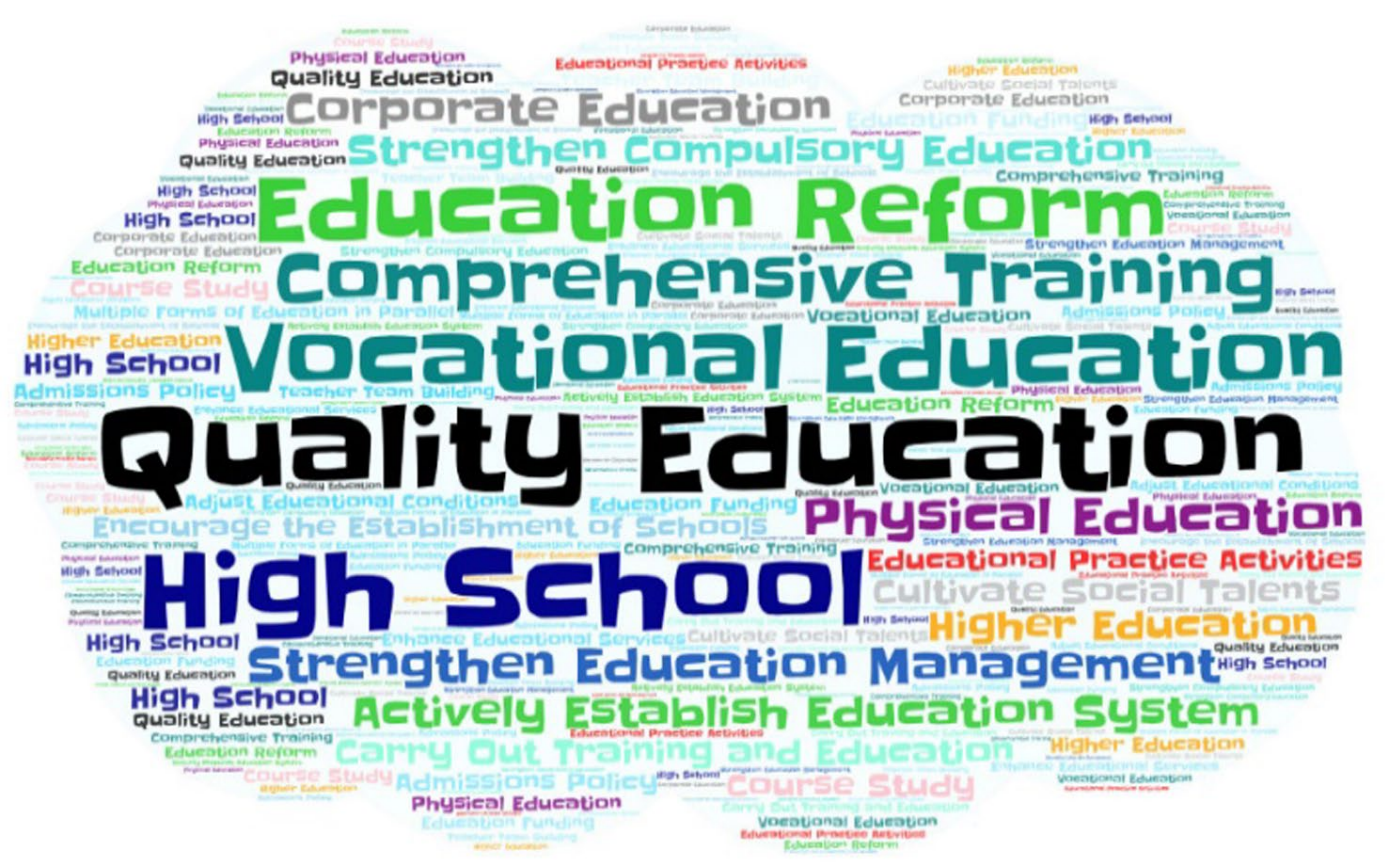
The word frequency map of the policy in encouraging development stage.

To cultivate new socialist talents that can meet the needs of modernization in the 21st century, China promulgated the “Decision of the Central Committee of the Communist Party of China and the State Council on Deepening Educational Reform and Comprehensively Promoting Quality Education” in 1999. Concrete measures were put forward in four aspects including promoting quality education, intensifying education reform, optimizing the structure, and strengthening the leadership. The “Decision” clearly put forward the issue of reducing the schoolwork burden of primary and secondary schools. Governments at all levels were tasked with establishing and improving the supervision and inspection mechanism to reduce the schoolwork burden on students. At the same time, it was actively encouraged and supported to run schools by social forces in various forms to meet the growing educational needs. Any school-running form that conformed to relevant national laws and regulations could be boldly tested. Preferential policies (such as preferential use of land and exemption from supporting fees) were to be formulated according to local conditions to support social forces in running schools (Yu et al., 2017). Shadow education beyond school education was not mentioned or strictly regulated. The word frequency diagram of the policy text at this stage is illustrated in Figure 2. From the perspective of the content of the policy text, “Quality Education,” “Physical Education,” and “Cultivate Social Talents” were mainly focused on. The policies called for cultivating all-around talents at this stage, but with quality education at the center, and advocated “Comprehensive Training.” The purpose of “High Schools” was to vigorously encourage rural schools to actively build several local colleges and universities. “Vocational Education,” “Compulsory Education,” “Various Forms,” and “Educational Funds” vigorously advocated various ways to raise education funds and complete the goals and tasks of compulsory education.

The quality education policy at this time ignored shadow education in design and inadvertently made it a huge market. The main goal of the “burden reduction” policy was to focus on the time and intensity of learning in schools, and the supervision and restrictions on school evening self-study and vacation make-up classes were more stringent, basically curbing the phenomenon of “arbitrary charges” and make-up classes in schools in various places. The time spent studying and preparing for exams in school decreased, but the demand for learning to choose a better school was still high. To gain a greater advantage in the competition for higher education, parents sought supplementary services of shadow education outside the school. During this period, a large number of tutoring institutions emerged and entered the stage of rapid market development.

### 3.3. Preliminary regulation stage (2010–2017)

With the rapid expansion of off-campus training institutions, the lack of government supervision brought many problems, and the lack of qualified individuals or “small workshop” tutoring institutions aroused widespread public opinion in society. The “burden reduction” policy did not achieve a significant effect. The academic burden of primary and secondary school students was transferred from on-campus to off-campus. Training institutions met the needs of families with the financial ability to obtain off-campus tutoring, and also greatly increased the financial burden of families (Zhang and Bray, 2015). The Outline of the National Medium and Long Term Educational Reform and Development Plan (2010–2020) clearly stated the “administration of private education by the law” and “regulate various social tutoring institutions and teaching aid markets.” To aid the implementation of the policy, The Training and Education Professional Committee of the Private Education Association was established in October 2010. In addition, the first “Self-discipline and Integrity Pact” for China’s training and education industry was issued to consciously maintain the industry order. The word frequency in the policy texts at this stage is shown in Table 3. “Educational Development,” “Normative Education,” “Educational Reform,” and “Establish Mechanism” strongly advocated reform and development with the view that only reform can promote the further development of education. “Education and Training,” “Private School,” and “Vocational Education” represented more training at this stage, and a series of problems emerged in shadow education. During this period, the state promulgated many laws and policies to further regulate off-campus training (Zhang and Bray, 2021).

**Table 3:** Statistics of high-frequency policy words in the preliminary regulation stage.

High-frequency words	Numbers	High-frequency words	Numbers	High-frequency words	Numbers
Educational development	175	School policy	98	Private school	76
Education reform	123	Vocational education	87	Normative education	65
Compulsory education	108	Education and training	78	Establish mechanism	58

The “Implementation Opinions of the Ministry of Education and Other Five Departments on the Work of Regulating Educational Fees and Governing Unauthorized Fees in Education in 2014” actively responded to the education issues that people were concerned about. In October 2016, the Chinese Society of Education promulgated the “Professional Standards for Teachers in Tutoring Institutions (Primary and Secondary Schools) (Trial)” to further standardize the market of off-campus training as well as the composition of teachers in tutoring institutions. In 2017, the “Opinions on Deepening the Reform of the Education System and Mechanism” specified that the training scope and content of off-campus training institutions needed to be standardized, and their school-running qualifications required strict review.

### 3.4. Comprehensive rectification stage (2018 to present)

**Figure 3:**
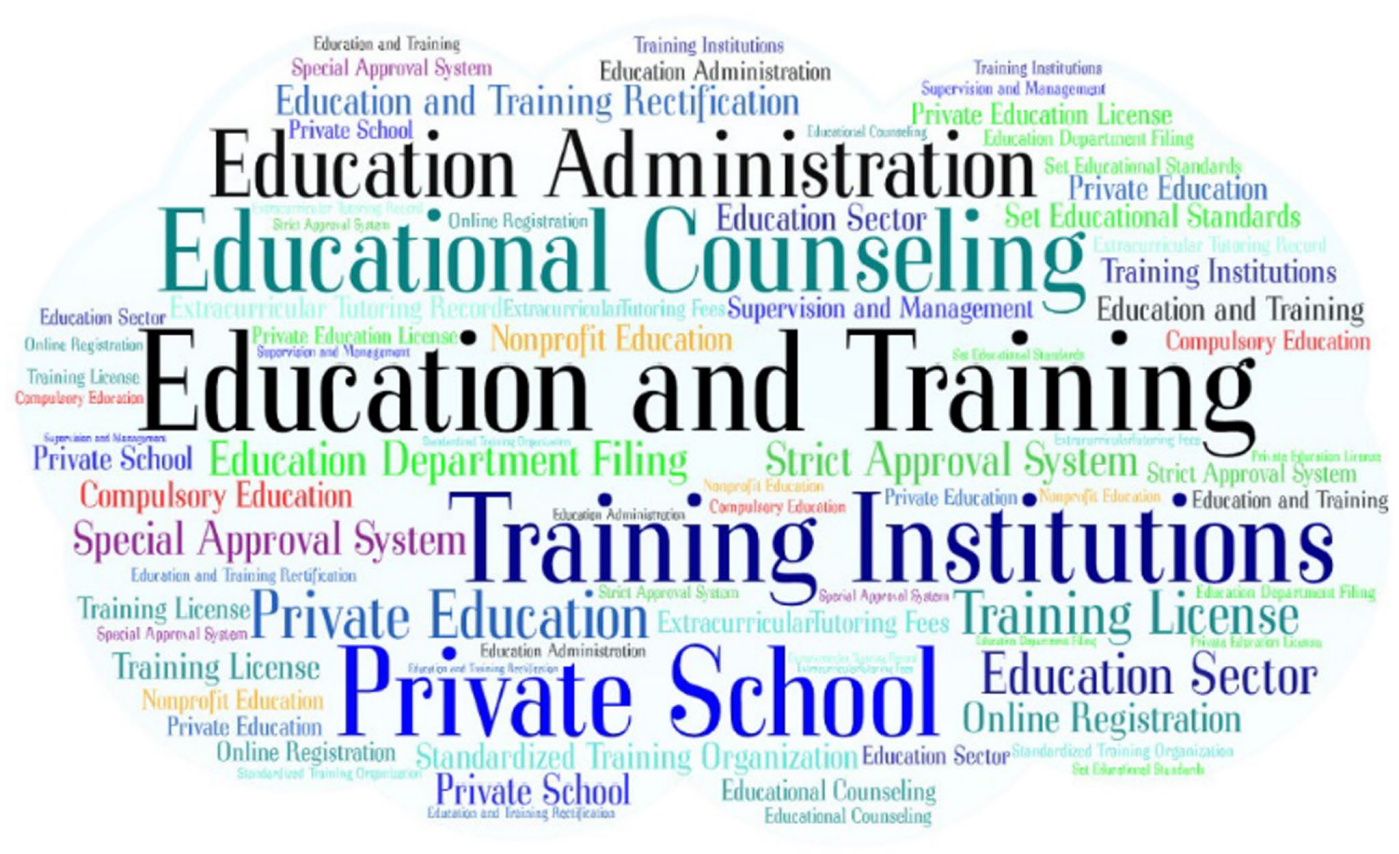
Policy word frequency map in the comprehensive rectification stage.

In February 2018, the General Office of the Ministry of Education and other four departments issued the “Notice on Effectively Reducing the Extracurricular Burdens of Primary and Secondary School Students and Carrying out Special Governance Actions for Off-campus Training Institutions,” which directly targeted off-campus training institutions to establish special governance and opened the prelude to the “burden reduction.” Shadow education governance entered a stage of comprehensive rectification. Subsequently, the Ministry of Education, the State Council, and other national competent authorities successively issued the “Notice on Accelerating the Promotion of the Special Governance of Off-campus Training Institutions,” “Opinions on Regulating the Development of Off-campus Training Institutions,” “Notice on Effectively Doing a Good Job in the Special Governance and Rectification of Off-campus Training Institutions Work,” “Notice on Improving Several Working Mechanisms for Special Governance and Rectification of Off-campus Training Institutions,” and other policy documents within 1 year. In August 2018, the General Office of the State Council issued the “Opinions on Regulating the Development of Off-campus Training Institutions,” which was the first systematic document in China to regulate the development of off-campus training institutions at the national level. Thus, the supervision of China’s shadow education entered a new era with laws and regulations to follow. The word frequency of the policy text at this stage is shown in Figure 3. During this stage, the shadow education policies that were issued became more specific and targeted. “Education and Training,” “Training Institutions,” “Private Schools,” and “Education Sector” appeared more frequently in the policy texts, the reason being that the policy at this stage directly aimed to rectify off-campus training. China has concentrated on social resources to rectify off-campus training, aiming to achieve the effect of “double reduction” and reduce the burden on primary and secondary schools. The appearance of terms such as “Training License,” “Strict Approval System,” “Education Administration,” and “Supervision and Management” demonstrate the determination of the Chinese government to take a drastic and comprehensive rectification of off-campus training.

All provinces across the country issued special governance plans for off-campus training institutions, clarified the timetable and roadmap for special governance, and comprehensively carried out “pull-net-type” thorough investigations. Further rectifications were carried out while examining and arranging. As of 12 December 2018, a total of 400,000 off-campus training institutions were surveyed nationwide, and 273,000 institutions were discovered with problems, 270,000 of which were rectified with a rectification completion rate of 98.9%. The General Office of the Central Committee of the Communist Party of China and the General Office of the State Council issued the “Opinions on Further Reducing the Burden of Students’ Homework and Off-campus Training in Compulsory Education,” which once again attracted great attention from the society. The Ministry of Education issued a notice to further clarify the scope of off-campus training disciplines and non-disciplines in the compulsory education stage to guide localities to do a good job in the governance of off-campus training (including online training and offline training). The bimonthly notification system and exposure platform for the situation resumed and a new round of special rectification work for off-campus training institutions began.

Reviewing the four stages of shadow education governance policies, it can be noticed that the government’s attitude toward the development of shadow education is different at different periods. It began with being acquiescent and then shifted to encourage the development of shadow education. Then, it turned to regulating and finally governing shadow education comprehensively. The introduction of government policies directly affected the development of shadow education, and analyzing the keywords helped distinguish the changes in the focus of the policies in different periods. In the first stage, the government acquiesced in the development of shadow education. Although it advocated reducing the burden on students, the implementation was weak. In the second stage, the focus on cultivating comprehensive talents and encouraging social forces to run schools resulted in vitalizing shadow education. In the third stage, education reforms promoted the preliminary regulation of the chaos in shadow education governance. In the last stage, rectification, standardization, and supervision became the mainstream policy focus which began to comprehensively rectify shadow education. Talent training and improvement of quality education have persistently been the goal of education development according to the analysis of the keywords. Although reducing the burden on students has been advocated in all the stages, the intensity of regulating shadow education differed in different periods. To analyze the reasons behind these changes in the shadow education policies over time, the multiple streams theory was used.

## 4. Multiple streams analysis of the evolution of China’s shadow education government policy

**Figure 4:**
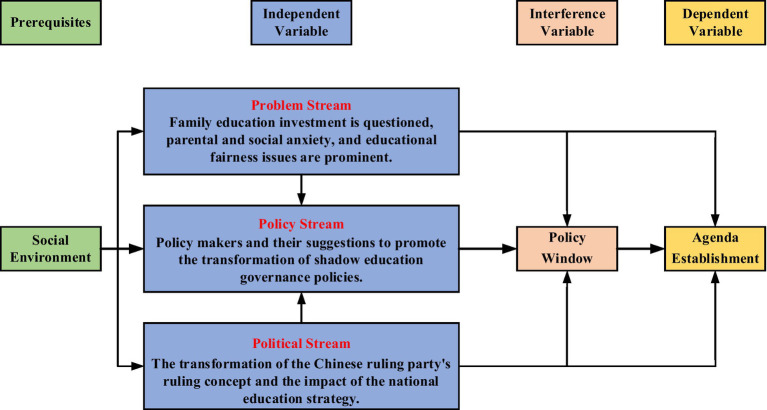
Multiple streams analysis framework of China’s shadow education policy evolution.

John Kingdon proposed a multiple streams analysis framework, which helped identify the reasons behind why the government paid attention to certain policies (Sabatier and Weible, 2014). Scholars have also proposed that the policy process is neither linear nor did it move at incremental stages (Smith and Larimer, 2018). The multiple streams theory has strong explanatory and predictive power and therefore was selected to analyze the introduction of the various shadow education governance policies over time as different streams played different roles in the introduction of the policies at different stages. There are large differences in the focus of policies in different periods, which leads to the question, what streams prompt the government to formulate relevant policies? Figure 4 illustrates the multiple streams analysis framework that was used to analyze the reasons for the evolution of China’s shadow education policy.

The first stream is the problem stream and refers to the “focusing incident” that can attract the attention of the public or government. Shadow education directly leads to social problems such as the vicious cycle of family education investment, the anxiety of parents and society, and education fairness. Then there is the political stream, which refers to the change in national ideology. To a certain extent, politics determines whether the problem will gain the required attention. Under the strategic guidance of the Chinese education department, the resources for primary education to higher education are inadequate, resulting in the extreme imbalance of resources at different schools. The last is the policy stream, which is composed of a series of policy solutions. Of course, it is also the choice of the policy solution to solve new problems. The changes in the understanding of policymakers on shadow education also affect the governance of shadow education. Proposals for education governance also impact policies governing shadow education.

### 4.1. Problem stream

The reasons why specific problems attract the attention of government policymakers can be determined through monitoring indicators that assess the importance of the problem and its changes and focus events or crisis events that drive the attention of the government and people around it and through feedback on the implementation of existing policies. The evolution of China’s shadow education governance policy has always been accompanied by changes in the problems to be solved. But the governance effect has not been fundamentally achieved.

In the early days of the reform and opening up, the Third Plenary Session of the Eleventh Central Committee of the Communist Party of China made major decisions centered on economic reconstruction. In the primary stage of the development of the socialist market economy, the educational sector accomplished obvious achievements such as resuming the college entrance examinations and promoting the development of compulsory education. However, there was still a big gap in the development requirements at that time. The rapid economic development urgently needed a large supply of talent. However, the educational resources at that time were seriously insufficient. There were great differences in the investment and distribution of educational resources. China’s education was basically in the stage of literacy and universal primary education. The method of selecting talents through examinations allowed the nascent development of shadow education.

With the promulgation of the decision to comprehensively promote quality education, various forms of schools functional at that time were encouraged to meet the growing educational needs while promoting quality education. The on-campus tutoring classes were gradually increased and off-campus training in various forms emerged. Before the year 2000, the cost of supplementary courses in schools continued to increase, which was closely related to the specific educational development situation during this period and the absence of government regulatory policies. After some time, China strictly prohibited on-campus tutoring, especially those charging a fee. Given that a huge gap between supply and demand still existed, extracurricular tutoring shifted from on-campus to off-campus. Private tutors and “small workshop” tutoring units evolved onto formalized large-scale institutions.

With the rapid development of tutoring institutions, a large number of problems emerged in the education market, which first attracted the attention of the state and the government and later led to heated discussions among the public and the emergence of strong public opinion. One such problem was the development trend of education industrialization. Data from the China Society of Education shows that the market size of tutoring in primary and secondary schools exceeded RMB¥ 80 billion in 2016, with 137 million students tutoring after class, and 7 million to 8.5 million teachers participating in tutoring institutions. For instance, in 2014, the students who participated in extracurricular tutoring classes in China accounted for about 36.7% of the total number of students in schools. In big cities such as Beijing, Shanghai, Guangzhou, and Shenzhen, it was as high as 70%. At the end of 2018, the General Office of the Ministry of Education notified the progress of the rectification of the special governance action for off-campus training institutions across the country. A total of 401,050 off-campus training institutions were surveyed nationwide, and 272,842 institutions had problems. After the special rectification action, the total number of off-campus training institutions increased to a peak of nearly 600,000 in 2019. In 2020, more than 400,000 were added and more than 100,000 were canceled. The total number of institutions far exceeded the number of schools in the compulsory education stage in the same period. While the large-scale growth of off-campus training institutions is still being brought under the purview of policy and governance, the accompanying problems such as advanced teaching, academic burden, and high costs have attracted great attention from society. The main motivation behind the development of off-campus training institutions is the strong market and the benefits of extracurricular tutoring, the development of marketization, and the power of capital. The main goal is to pursue economic growth. The scale and speed of its industrialization and development pose greater challenges to school education. The test-oriented teaching that blindly seeks scores has seriously damaged the educational ecology (Zhang and Bray, 2018).

The second is the vicious circle of family investment in education. With the increasing importance of education in the talent selection process, academic status is a stepping stone to obtaining social positions and social status. School education cannot meet students’ needs for higher education resulting in the rapid development of shadow education. Parents who are dissatisfied with the school’s main education turn to off-campus training institutions to make up for it and to gain a competitive advantage. According to the “2017 China Education Finance Household Survey (CIEFR-HS)” data released by the China Institute of Educational Fiscal Science of Peking University, the total expenditure of household education at the basic education stage nationwide is about RMB¥ 1904.26 billion, accounting for 2.48% of the GDP in 2016. The national average household education expenditure per student at the basic education stage is RMB¥ 8,143. In 2016, China’s fiscal education expenditure exceeded RMB¥ 3 trillion, while the family education expenditure reached nearly RMB¥ 2 trillion. China’s off-campus training market is as high as RMB¥ 800 billion, which is a vivid illustration of the family education expenditure of nearly RMB¥ 2 trillion.

The third is parental anxiety and social anxiety. The differences in educational resources make Chinese parents anxious, and pass this educational anxiety to their children, extending to primary and secondary schools and even kindergartens (Zhang, 2020). Under the current social education background in China, the more the parents’ attention is paid to education, the higher their educational anxiety. The focus of reducing the extracurricular burden of primary and secondary school students is to not only rectify off-campus training institutions but also reduce the educational anxiety of parents. The reason for the extracurricular burden of primary and secondary school students is inseparable from the educational anxiety of their parents. The most direct manifestation is to give children extracurricular tutoring and carry out supplementary education or advanced education. The theater effect behind the Huangzhuang craze is spreading in Chinese education. Off-campus training under the theater effect has intensified. Crazy cram schools and after-school homework have devastated teenagers physically and mentally. In the high-pressure learning state, teenagers have frequently suffered from psychological problems and suicidal intentions. It violates the rules of talent training and runs counter to the original intention of educating. Parents involved in the theater effect are anxious and suffer from serious anxiety. The statistics of public opinion data on education and training consumption in 2020 by the Beijing Sunshine Consumption Big Data Research Institute and Consumer Network show that, of the 3,847,566 entries of public opinion on education and training consumption, 2,712,138 were negative, accounting for 70.49%.

### 4.2. Political stream

To explain the changes in China’s shadow education governance policy, it is important to discuss the perspective of education development from the dimension of the political stream. The change in the ruling party’s concept of governance has an obvious promoting or inhibiting effect on the influence of the policy agenda.

In the early days of reform and opening up to “produce talents quickly and produce good talents,” the Party and the state have concentrated their efforts to run several key universities, middle schools, and primary schools, where the funds, school conditions, and student resources were uneven. As a result of rigorous examinations, the best talents were concentrated in key schools. Key schools needed to be restored not only in colleges and universities but also at primary and secondary school levels. In January 1978, the Ministry of Education pointed out in the “Implementation Plan on Running a Batch of Key Primary and Secondary Schools”: “We must conscientiously run a batch of key primary and secondary schools to facilitate the experiment of educational revolution, summarize the practical experience, and promote the education reform in depth in primary and secondary schools.” In 1995, China formally proposed the strategy of rejuvenating the country through science and education and launched the “211” project, and in 1998, it implemented the 21st-century education revitalization action plan, proposing to “deepen the reform of the school-running system and mobilize the enthusiasm of all aspects for educational development.” The pattern of “layers of priorities” in key schools was thus formed. The priority of resource allocation from primary education to higher education has resulted in an extremely uneven development among schools. For a long time, the national standard for talent selection has mainly been based on academic examinations. The “elitist” talent training and selection model has promoted the development of shadow education, and secondary vocational diversion has exacerbated parents’ anxiety and shadow education.

### 4.3. Policy stream

The policy stream is designed to analyze the process by which policy options are generated, discussed, revised, and valued. In terms of the policy evolution process of China’s shadow education governance, the policy stream mainly analyzes policymakers’ understanding of shadow education, and how experts and scholars “soften” the shadow education governance ideas at different stages in the policy changes of shadow education governance.

First, changes in policymakers’ perceptions of shadow education affect the focus of shadow education governance. Since the start of the reform and opening up, relevant policymakers’ perceptions of China’s shadow education have changed four times. For the first time, in the early stage of reform and opening up, China’s education was in a period of order restoration. In the case of a relatively serious shortage of educational resources, the ideological and subconscious thought was that shadow education in the form of tutoring could make up for the insufficiency of school education and would not affect mainstream education. Therefore, no attention was paid to the existence of shadow education in its nascent stage. The second time happened with the rapid development of society and the economy, and various forms of running schools emerged to meet the needs of the people for education. A large number of off-campus training institutions emerged against the background of market-oriented development. Given that schools were required to reduce the academic burden of students, various training forms expanded to meet the all-around development of quality education. The third time was when many problems emerged after the rapid expansion of off-campus training institutions. The attitudes of policymakers changed on shadow education governance, and they began to pay attention and face the problems and supervised the off-campus training market through industry self-discipline and norms. The fourth time was at the beginning of 2018 when the public began to demand fairness in higher-quality education with the development of education in China. The continuous expansion of shadow education despite regulation threatens the existence of the main education system. The trend in education industrialization and capitalization has compelled policymakers to issue policy directives with the aim to solve the problems.

Second, the recommendations of experts and scholars provide a blueprint for policy options, especially the proposals on shadow education governance to promote changes in shadow education governance policies. During its two sessions each year, the National People’s Congress delegation actively offered advice and suggestions on the problems existing in China’s examination-oriented education, and the proposals on burden reduction and education reform were taken seriously, which prompted the introduction of a series of shadow education governance policies since 2018.

### 4.4. Policy window opens

The objectives, scope of adjustment, and protection of rights and interests of China’s shadow education governance policy have undergone significant changes over time. From the perspective of the multiple streams theory, the changes in the problems to be solved in shadow education governance drew attention to it and provide a rational basis for policy changes; the transformation of national strategies in the political stream created a favorable political environment for changes; and policy streams converged with the policy community providing suggestions and encouraging policymakers to adopt them, forming a legal basis for change. Through continuous interaction, brewing, and interweaving, the three sources jointly promoted the opening of the window of opportunity for policy change. In most cases, the window was opened because of the problem and the political stream. The policymakers could recognize the opportunities brought by the window and build on the three source flows. A confluence of these brought about policy change in a short period of time.

In the first stage, shadow education appeared and developed slowly. At this stage, the field of education gradually resumed, and an important mechanism of selection based on examinations was established. Coupled with the rapid economic development, affluent families increased the demand for extracurricular counseling, thereby opening the window of shadow education to acquiescence in this stage. In the second stage, on-campus supplementary courses turned to off-campus supplementary courses, and training institutions developed rapidly. At this stage, the government promoted quality education and deepened education reform, and promulgated a number of policies to encourage social forces to run schools. The political and policy stream opened the window, making this stage encourage the development of shadow education. In the third stage, normative governance had not achieved effective results, and shadow education was still expanding on a large scale. Many problems surfaced, threatening the mainstream education system, and the education problems raised by society attracted great attention from policymakers. Shadow education governance had become more important and urgent in education and people’s livelihood. To solve the social problems brought by shadow education at this stage, the problem streams directly opened the window of policies, making this stage the preliminary standard stage of shadow education. In the fourth stage, on the one hand, social problems brought by shadow education, in reality, had not been resolved. On the other hand, the state had politically required strict control of the development of shadow education, and a series of governance policies had been introduced. This left the three sources highly in sync to promote the release of the “Opinions on Regulating the Development of Off-campus Training Institutions” policy; a series of policies have since been introduced receiving great attention from the whole society and will continue to govern shadow education until the problem is effectively solved.

Compared with the stream of problems and the stream of policies, the political stream, especially the transformation of the existing governance concept, has played the most important role in the introduction of policies, especially the transformation of China’s shadow education governance policy to a mandatory normative approach.

## 5. Optimization of shadow education governance policies from the perspective of multiple streams

### 5.1. Improve the quality of education in public school

The education quality of public schools directly affects the chances of students participating in extracurricular counseling. When public schools cannot meet the demands of students and parents, they will turn to shadow education. It is difficult for teachers to take into account the learning situation of each student, especially in a large class teaching setup. Under the assessment and reward mechanism, teachers are more inclined to focus on training students with better grades. On the contrary, students with poor grades lack enough attention. As a result, it is difficult for students with poor grades to make up for the lack of understanding of specific subjects in a short time. However, some teachers in public schools have poor teaching abilities and are not able to promote the improvement of students in the middle and upper levels, resulting in parents trying to make up for lessons for their children. Therefore, the optimization efforts of shadow education governance policies should improve the teaching quality of public schools, improve the level of teaching teachers, adopt small class systems, pay attention to the learning and living conditions of each student, and reform the evaluation mechanism of public schools. Education should meet students’ personalized requirements as far as possible. Teachers must keep learning and enriching themselves so as to meet the requirements of teaching, such as simplifying complex issues and focusing on cultivating students’ thinking. Teachers may develop customized counseling plans for them according to the students’ strengths and weaknesses. Second, public institutions should comprehensively improve the integrity and timeliness of educational output, adhere to the requirements of comprehensive quality and educational modernization with strict teaching standards, and improved the quality of teaching. Finally, schools should learn from extracurricular tutoring education institutions to analyze their own shortcomings so that they can curb the development of shadow education to a certain extent.

### 5.2. Systematic planning and phased improvement of the shadow education governance policy system

China’s shadow education governance is a policy choice based on the problem of “reducing the burden.” The government’s motivation for shadow education governance and its understanding of the nature of shadow education governance is based on reducing the academic burden of students (Zhang and Bray, 2017). At present, China’s shadow education governance policy is mainly based on limiting shadow education, which solves the problem of capitalization of the education industry in a timely and effective manner to a certain extent. However, shadow education is a unique policy situation accompanying the formation of China’s basic education system, and its governance needs to be placed within China’s entire education system and social field. As part of the reform and development of the national education system, shadow education governance is long-term and complex and needs to be coordinated with a holistic concept. The first step is to scientifically design a diversion system that conforms to the laws of education and change the existing junior high school diversion system to high school diversion. The industrial division of labor and technical requirements in modern society is more complex and the requirements for the comprehensive quality of laborers are more stringent. Talents along with the comprehensive quality of ordinary high school graduates have more development potential. The reform of the secondary school diversion system can effectively alleviate the educational anxiety and pressure transmitted to the basic education stage. High school graduates are adults and can choose their own university studies, so parents are willing to leave the choice to the students themselves. The second step is to reform the existing college entrance examination admission system, further improve the system from the source of the higher education selection mechanism, and select various talents in a diversified way. The natural stratification of higher education resources leads to the fact that no matter how ingenious the improvement plan of educational resource allocation is, students can only compete within the same track, and cannot fundamentally give up their enthusiasm for participating in shadow education. The third step is to improve legislation and strengthen legislative provisions on off-campus training.

### 5.3. Multi-party subject participation solves the real problem of shadow education governance

Against the background of “double reduction,” China currently restricts the supply end and prohibits advanced training of disciplines, which curbs the excessive demand for extracurricular tutoring by families in the short term. However, it cannot effectively curb the popularity of tutoring in the long run. If there is no systematic reform of the education system, limiting the supply of tutoring alone will simply delay the level of education that may occur after extracurricular tutoring. In the mechanism of selecting talents by examinations and scores, the demand for preparation time still exists. Parents will still choose to increase spending or hire high-salary tutors to meet the demand. Off-campus tutoring will become more hidden and more expensive, making it difficult for the average family to pay. Government regulation will also become more difficult, possibly creating new unequal educational opportunities. From the perspective of game theory, this highlights the existence of game competition between the government and extracurricular tutoring enterprises (Li et al., 2022). To completely solve the internal “heat” of off-campus training, China must correct the educational reform logic from the root and internal and external aspects, that is, correct the utilitarian tendency of education reform from the outside, and at the same time adhere to the human-centered position of education from the inside. Only this solution can address both the symptoms and the root causes, promote the benign coexistence of shadow education and school education, and protect the vigorous growth of primary and secondary school students. Given that the current education system has not been fundamentally reformed, to gain a competitive advantage, off-campus training will once again evolve into private tutoring, exacerbating inequality in educational opportunities. The question of who will make up for canceled after-school services will be a new issue under the existing policy. Therefore, the shadow education governance policy is a long process. It should build an excellent resource-sharing platform. The government should incorporate shadow education into the basic national education system to play a part in big data analysis ability and innovation of business models (Cui et al., 2022). Of course, shadow education institutions should increase public welfare investment and help students with poor family backgrounds, such as qualified students can access it at lower costs (or free). To truly solve the problems of shadow education and address the learning and academic problems faced by students, it is urgent for schools, families, the government, and society to coordinate and improve after-school services.

## 6. Conclusion

Shadow education is widely prevalent in countries around the world, and the governance policies concerning them differ considerably. As a country with significant shadow education, China has pursued different policies toward shadow education at different stages. Therefore, the focus of this article was to trace the evolution of China’s shadow education governance policy and examine the basis of the introduction of the governance policy with the background of the “double reduction” policy. To better analyze the historical changes in China’s shadow education, as a first step, 44 policy texts from 1978 to 2022 were categorized under four stages according to the key content promulgated by the policy. Second, text mining was used to analyze shadow education governance policies in different periods. Using the Python software to obtain high-frequency vocabulary that appeared in different periods of the policy texts, the emphasis on the shadow education governance policies at different stages was analyzed to compare the differences. An in-depth analysis of the reasons for the promulgation of the policy was also undertaken. Then, to further understand the reasons for the promulgation of China’s shadow education governance policy, this article used the theoretical analysis method of multiple streams theory and analyzed the factors that affected the changes in shadow education governance policies from the perspectives of problem stream, policy stream, and political stream. These analyses were undertaken to find ways to improve the governance of shadow education. Finally, to address the gaps in China’s current shadow education governance policy, relevant recommendations were put forward.

The results of the study found that, first, successive government policies have not shifted the focus from reducing the burden to promoting fairness in education and have failed to form effective measures to ensure educational fairness. Second, a comparison of the outcome of the governance policies reveals that the transformation of the political stream, especially the ruling party, played the most important role in the introduction of policies. Third, contradictions and conflicts persist in the governance policy of shadow education at different periods.

Therefore, to optimize the governance policy of Chinese shadow education, it is necessary to fully consider and ensure the participation of different classes in the process of shadow education governance, especially the low-income groups in shadow education to avoid aggravating class solidification. Second, there is a need to improve the social status and treatment of vocational and technical personnel and truly realize fair competition between different types of education. Third, efforts must go into incorporating shadow education into the governance of building a strong country, promoting education fairness, and forming a supply of high-quality talents. Fourth, the shadow education governance policy promulgated by different departments needs to be coordinated and cooperated. In particular, it is necessary to introduce shadow education governance policies tailored to local conditions according to the actual situation and comparison of different regions (Zhang and Bray, 2020), summarize the effects and deficiencies of past policies, and continuously improve the shortcomings of policies. Finally, there is a need to actively advocate the work idea of the “double reduction” policy so that teachers, parents, and students can actively give up extracurricular tutoring and seize the efficiency of classroom learning at school, thereby truly reducing the burden on students and achieving educational equity to a certain extent.

## Author contributions

KX and JL: conceptualization, methodology, formal analysis, and review and editing. KX: investigation and original draft preparation. XC and AZ: supervision and funding acquisition. All authors contributed to the article and approved the submitted version.

## Funding

This work was supported by the National Social Science Foundation of China (Major Project No. 19ZDA066).

## Conflict of interest

The authors declare that the research was conducted in the absence of any commercial or financial relationships that could be construed as a potential conflict of interest.

## Publisher’s note

All claims expressed in this article are solely those of the authors and do not necessarily represent those of their affiliated organizations, or those of the publisher, the editors and the reviewers. Any product that may be evaluated in this article, or claim that may be made by its manufacturer, is not guaranteed or endorsed by the publisher.

## Footnotes

^1^ http://www.bailuzhiku.com/
